# The Influence of pH on the Catalytic Capacity of Levodopa in the Electroreduction Processes of Zn^2+^ Ions

**DOI:** 10.3390/molecules30122590

**Published:** 2025-06-13

**Authors:** Jolanta Nieszporek, Tomasz Pańczyk

**Affiliations:** 1 Institute of Chemical Sciences, Department of Analytical Chemistry, Faculty of Chemistry, Maria Curie-Sklodowska University, M. Curie-Sklodowska Sq. 3, 20-031 Lublin, Poland; 2 Jerzy Haber Institute of Catalysis and Surface Chemistry, Polish Academy of Sciences, ul. Niezapominajek 8, 30-239 Cracow, Poland; tomasz.panczyk@ikifp.edu.pl

**Keywords:** levodopa, zinc ions, electroreduction, inhibition, acceleration, kinetics

## Abstract

The aim of the study was to investigate the influence of L-DOPA—the gold standard in the treatment of Parkinson’s disease symptoms—on the electroreduction kinetics of Zn^2+^ ions. It was demonstrated that this effect depends not only on the concentration of the drug but also on the environment in which the process takes place. In the experimental part, cyclic voltammetry (CV), square wave voltammetry (SWV), direct current polarography (DC), and electrochemical impedance spectroscopy (EIS) were used. Based on the obtained results, it was determined that the analyzed electrode reaction, both in the absence and presence of L-DOPA, proceeded in two steps. The kinetic parameters of Zn^2+^ ion electroreduction indicated its quasi-reversible nature in solutions with both pH = 2.0 and pH = 6.0. The presence of the drug in the lower pH solution resulted in a slight slowing down of the electrode process, whereas in the pH = 6.0 solution, it led to a significant acceleration. In both low and high pH solutions, the first step was slower and determined the rate of the entire electrode process.

## 1. Introduction

Parkinson’s disease is a neurological disorder that is associated with the gradual depletion of dopamine-producing neurons [1]. It is characterized by the following symptoms: loss of balance, muscle tension, tremors of the limbs at rest, slowed body movements, and impaired cognitive functions [2]. This neurodegenerative process causes changes in the homeostasis of neurotransmitters and metal ions, such as zinc [3]. The gold standard in the treatment of motor symptoms of Parkinson’s disease is levodopa (L-DOPA), which works by supplementing dopamine levels in the brain [1]. Studies involving L-DOPA indicate its interactions with metal ions [4]. Thanks to this, the use of metal chelates, e.g., L-DOPA–Zn^2+^ instead of high doses of L-DOPA itself, allows for lower and optimal dosing and the avoidance of the drug’s side effects [5].

**Figure 1:**
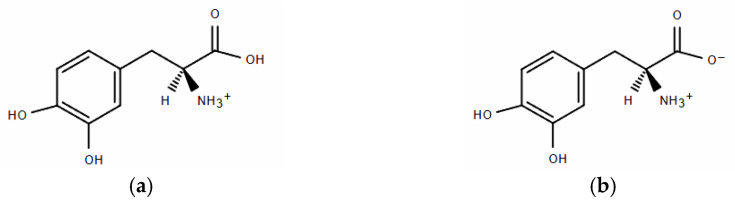
(**a**) The cationic form of L-DOPA. (**b**) The zwitterion form of L-DOPA.

The form of drug administration is primarily determined by the patient’s condition. There are oral and parenteral routes. To obtain the best bioactivity of levodopa, it is important to understand its molecular state in the skin, gastrointestinal tract, and blood. Studies indicate that gastric pH is highly acidic (range 1.0–2.5), pH in the proximal small intestine is 6.6, normal skin surface pH stays between 4 and 6.5, and blood pH is regulated to stay within the narrow range from 7.35 to 7.45 [6]. It has been shown that the levodopa molecule appears in different ionic forms, depending on pH [6,7]. At low pH values below 2.3, the amino group is protonated. Consequently, in significantly acidic solutions, levodopa occurs in two ionic forms: cationic and as a zwitterion (Figure 1a,b). When the pH reaches values of 2.3–8.11, the levodopa molecule occurs only in the zwitterion form (Figure 1b) [8].

Studies involving levodopa have often been conducted in terms of its detection and characterization of electrochemical behavior [9,10,11]. Yan et al. showed that L-DOPA is irreversibly oxidized on glassy carbon electrodes, and the use of single-wall carbon nanotube-modified glassy carbon electrodes was significantly improved. Moreover, it turned out that the oxidation of L-DOPA at the modified electrode was a diffusion-controlled process [12]. Socorro et al. conducted cyclic voltammetry measurements vs. Ag/AgCl and showed a redox couple with anodic and cathodic peak potentials at 0.58 V and 0.52 V, respectively [13].

It is also necessary to emphasize the invaluable importance of the aforementioned zinc as a microelement. It has an extraordinary ability to bind to ligands that are easily exchangeable with organic molecules, including proteins and nucleic acids [14]. It is this ability of zinc that underlies its inclusion in many basic metabolic processes of cells. The tertiary structure of many proteins is determined by zinc ligands. For this reason, Socorro et al. conducted cyclic voltammetry measurements vs. Ag/AgCl and showed a redox couple with anodic and cathodic peak potentials at 0.58 V and 0.52 V, respectively [13]. The integrity of cell membranes and some ion channels depends on this microelement. The multitude of processes that depend on zinc also includes neuronal transmission in the central nervous system [15]. It is known that zinc homeostasis is changed in pathological conditions. Therefore, our studies on the effect of L-DOPA on the kinetics of the electroreduction of Zn^2+^ ions can be helpful in explaining the mechanism of drug action in Parkinson’s disease. Considering the fact that the mercury surface is hydrophobic like the cell membrane surface and that the mercury electrode potential values are similar to those on cell membranes, it seems that the systems selected for measurements are attractive. They can be treated as models for real ones occurring at the boundary of biological membranes.

Moreover, the presented results may be of interest from the perspective of research on steel corrosion inhibition. The fact that the L-DOPA molecule contains 3-hydroxyl, amine, and keto groups is very important for the organic compound to behave as a corrosion inhibitor. Sachin et al. [16] demonstrated the effective inhibition of mild steel corrosion by L-DOPA in hydrochloric and sulfuric acid solutions. The corrosion inhibition was explained by adsorption and the formation of an L-DOPA film on the metal surface. In turn, Yang et al. investigated the effect of poly-(L-DOPA) on the corrosion inhibition of carbon steel in HCl. Based on electrochemical measurements, they showed that with an increasing concentration of poly-(L-DOPA), the corrosion inhibition efficiency increased and could reach up to 97% [17]. Thus, the organic compounds containing nitrogen in their molecular structure, such as L-DOPA, can protect metal from the aggressive action of the environment. By adsorbing onto the metal surface, they slow down its corrosion.

In summary, L-DOPA is an extraordinary molecule that plays an important role in medicinal chemistry, pharmacology, and even protection from corrosion.

The objective of the studies was to determine the effects of pH and levodopa concentrations on the kinetics and mechanism of the electroreduction of Zn^2+^ ions. It should be noted that the process of zinc electrodeposition on the mercury electrode takes place in the range of negative potentials very far from the oxidation potential of L-DOPA. In this way, it makes it an electrode-inactive substance in the potential range of zinc ion depolarization. This is one of the conditions of the cap-pair rule that the organic substance-depolarizer ion system must meet in order to catalyze the electrode reaction. This rule is based on the statement that the catalytic effect is a consequence of the formation of an active complex on the electrode surface between the adsorbed organic substance and the depolarizer ions. The formed active complex facilitates the exchange of charge during electroreduction. The organic compound must therefore have ligand atoms, e.g., nitrogen or sulfur. This is another condition of the cap-pair rule. The rule also requires that the reduction potential of the depolarizer is within the range of the labile adsorption equilibrium of the organic compound on the mercury electrode. The rule explaining the acceleration of electrode processes was formulated by Sykut’s team [18].

## 2. Results

### 2.1. Adsorption Properties of Levodopa

**Figure 2:**
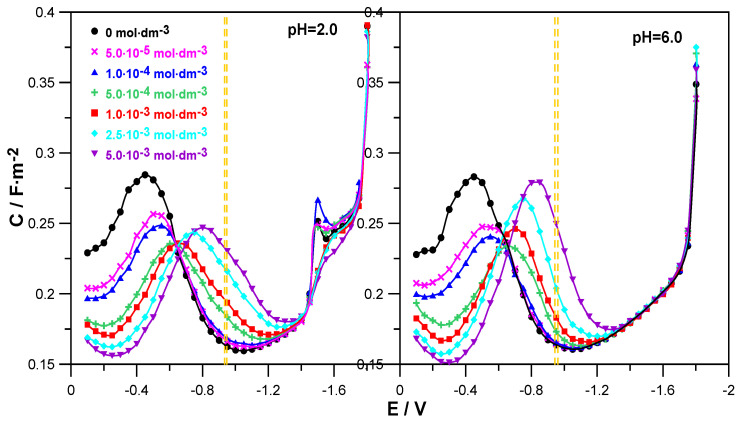
Differential capacity–potential curves of the mercury electrode in contact with 1.0 mol·dm^−3^ NaClO_4_ at pH = 2.0 and pH = 6.0 (⏺) + different amounts of L-DOPA indicated in the Figure. Additionally, the Figures show the range of values of the formal electroreduction potential of Zn^2+^/Zn ions in solutions with pH = 2.0 and pH = 6.0, determined in the absence and presence of L-DOPA, marked with yellow dashed lines.

Figure 2 shows the changes in the differential capacitance of the double layer as a function of the electrode potential for the adsorption of L-DOPA on mercury from 1.0 mol·dm^−3^ NaClO_4_ at pH = 2.0 and 6.0.

The comparison of the C = f(E) curves obtained in the presence of the drug in solutions at both pH = 2.0 and pH = 6.0 with the differential capacitance curve of the supporting electrolyte distinguished two areas of L-DOPA adsorption potentials. In the range of less negative potentials from E = −0.1 V to E ≈ −0.6 V, the presence of L-DOPA in the NaClO_4_ solution caused a clear decrease in differential capacitance in relation to that recorded for the supporting electrolyte. The higher the concentration of the organic compound, the greater it was and indicated an increase in the adsorption of L-DOPA on mercury. It is worth noticing that this effect was a bit clearer in the case of solutions with a pH = 6. This indicates a slightly stronger adsorption of L-DOPA at pH = 6.0. In the range of potentials −0.6 V to −1.4 V, the opposite situation was observed. An increase in the drug concentration caused a growth in the differential capacitance. This regularity applied to both solutions with pH = 2.0 and 6.0. Then, in the range of the most negative potentials E < −1.4 V, the C = f(E) curves converged, which indicated a lack of L-DOPA adsorption on mercury in this electrode potential range.

In the range of Zn^2+^ ion reduction potentials (Figure 2, the area marked with yellow dashed lines), the use of L-DOPA at concentrations of c ≤ 1.0 × 10^−4^ mol·dm^−3^ caused a slight increase in the differential capacitance in relation to the capacity of NaClO_4_. However, in the range of higher L-DOPA concentrations c ≥ 5.0 × 10^−4^ mol·dm^−3^, this increase was clear and grew with increasing amounts of the drug. Nevertheless, this was not the range of strong adsorption of L-DOPA on the mercury electrode but could rather be called the area of labile adsorption. In this way, one of the conditions of the cap-pair rule was met.

In addition, the analysis of the changes in the differential capacitance curves shown for the solution at pH = 2.0 enabled us to notice that at the potential E = −1.5 V for NaClO_4_, a small peak was formed, the height of which increased in the presence of the smallest amounts of L-DOPA (c ≤ 1.0 × 10^−4^ mol·dm^−3^). Further growth of the drug concentration caused a gradual extinction of this peak. The peak observed in the solution at pH = 2.0 was probably related to the electrochemical reduction of perchlorate ions [19].

### 2.2. Kinetics and Mechanism of Electroreduction of Zn^2+^ Ions in the Presence of Levodopa

**Figure 3:**
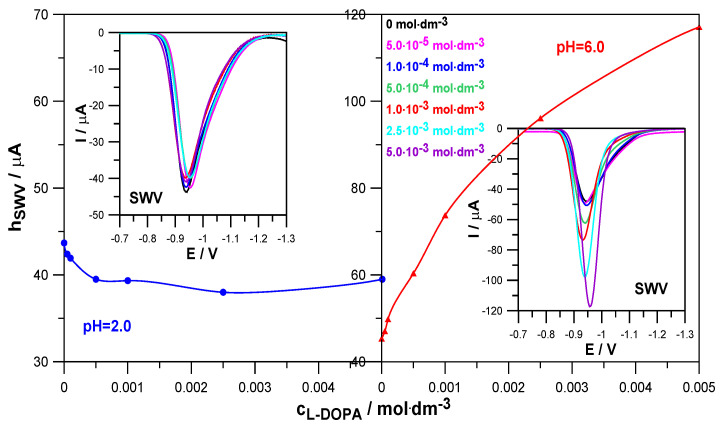
SWV peak heights as a function of L-DOPA concentration and SWV voltammograms of the electroreduction of Zn^2+^ ions in 1.0 mol·dm^−3^ NaClO_4_ and in the presence of L-DOPA at concentrations indicated in the Figure in solutions at pH = 2.0 and pH = 6.0.

Levodopa is an organic compound that is characterized by labile adsorption on the mercury surface in the range of Zn^2+^ reduction potentials (Section 2.1). It can therefore affect the rate of the aforementioned electrode process. The SWV voltammetry method allows for the qualitative assessment of whether and how the drug changes the kinetics of zinc electrodeposition on mercury. Figure 3 shows how an increase in L-DOPA concentration affects the height of SWV peaks in depolarizer solutions at pH = 2.0 and pH = 6.0.

In a solution with pH = 2.0, the presence of levodopa slightly reduced the height of the SWV peaks, compared to the height of the peak corresponding to the reference solution (Zn^2+^ in the absence of levodopa). This indicates a slight inhibitory effect of this compound on the rate of the electroreduction of Zn^2+^ ions. In a solution with pH = 6.0, a clear increase in the height of the SWV peaks and a decrease in the width of the peaks in the middle of their height were observed due to the increase in the concentration of levodopa. This proves its catalytic abilities on the kinetics of the analyzed electrode reaction [20,21]. Such different behavior in solutions with pH 2.0 and 6.0 probably resulted from the different forms in which levodopa occurred, depending on the pH.

**Figure 4:**
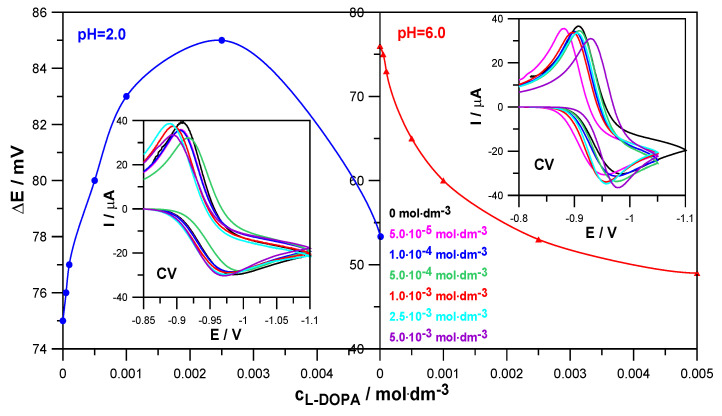
Values of the difference between the anodic and cathodic peak potentials ∆E = E_a_ − E_k_ on CV voltammograms as a function of L-DOPA concentration and CV voltammograms of the electroreduction of Zn^2+^ ions in 1.0 mol·dm^−3^ NaClO_4_ and in the presence of L-DOPA at concentrations indicated in the Figure in solutions at pH = 2.0 and pH = 6.0.

Similar conclusions were obtained based on the results of CV voltammetry measurements (Figure 4). The values of the difference between the anodic and cathodic peak potentials, ∆E = 75 mV at pH = 2.0 and ∆E = 76 mV at pH = 6.0, obtained for the electroreduction of Zn^2+^ in 1.0 mol·dm^−3^ NaClO_4_ in the absence of the drug indicate the quasi-reversible nature of this process. On the other hand, an increase in the concentration of levodopa resulted in a slight increase in the ∆E values in solutions with pH = 2.0 and a significant decrease in solutions with pH = 6.0. Such trends of changes confirm earlier conclusions about a slight slowing down of the electrodeposition of zinc on mercury by L-DOPA in solutions with a lower pH and an acceleration in solutions with a higher pH [22]. It can be seen that the catalytic effect of the drug is much more pronounced than its inhibitory abilities.

In addition, CV voltammograms allowed us to determine the values of reversible half-wave potentials and formal potentials of the electroreduction of Zn^2+^ ions in the analyzed systems [23]. Since their values changed only slightly, it can be concluded that stable Zn^2+^-levodopa complexes were not formed in solutions of pH = 2.0 and pH = 6.0 [24].

**Figure 5:**
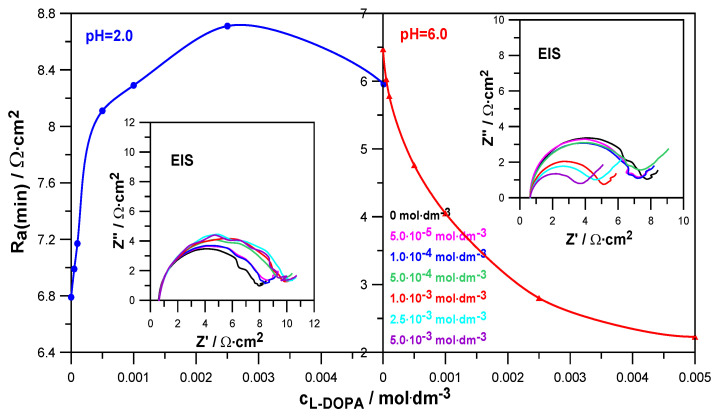
Values of the minimum charge-transfer resistance R_a(min)_ as a function of L-DOPA concentration determined from the EIS spectra in solutions with pH = 2.0 and pH = 6.0. Impedance diagrams measured at a formal potential for the electroreduction of Zn^2+^ ions in 1.0 mol·dm^−3^ NaClO_4_ at varied concentrations of L-DOPA.

The electrochemical impedance spectroscopy EIS method helps to determine the kinetics of electrode reactions and the characteristics of the electrode/electrolyte solution interface. Figure 5 shows the EIS spectra recorded at formal potentials for the electroreduction of Zn^2+^ ions in NaClO_4_ at pH = 2.0 and pH = 6.0. Based on them, the values of the minimum charge-transfer resistance R_a(min)_ were determined, which, as a function of L-DOPA concentration, are also shown in Figure 5. The decrease in the R_a(min)_ value in the presence of levodopa in solutions at pH = 6.0 indicates a clear catalytic effect of this compound on the electroreduction of Zn^2+^. Different behavior is shown by the dependence R_a(min)_ = f(c_L-DOPA_) obtained in a solution at pH = 2.0. The increase in the values of the minimum charge-transfer resistance caused by the increase in the drug concentration indicates the increasing difficulty of depolarization of Zn^2+^ ions on the mercury surface [25].

In order to precisely determine the effect of levodopa adsorption on the mercury electrode on the kinetics and mechanism of zinc electrodeposition, depending on the acidity of the solution, the electrode reaction rate constants k_f_ were determined for the analyzed systems as a function of the electrode potential. The following parameters were used for these calculations: charge-transfer resistance, reversible potential of the half-wave electrode reaction, and diffusion coefficients of zinc ions in the analyzed solutions. The details and calculation methodology were described in another paper [24].

**Figure 6:**
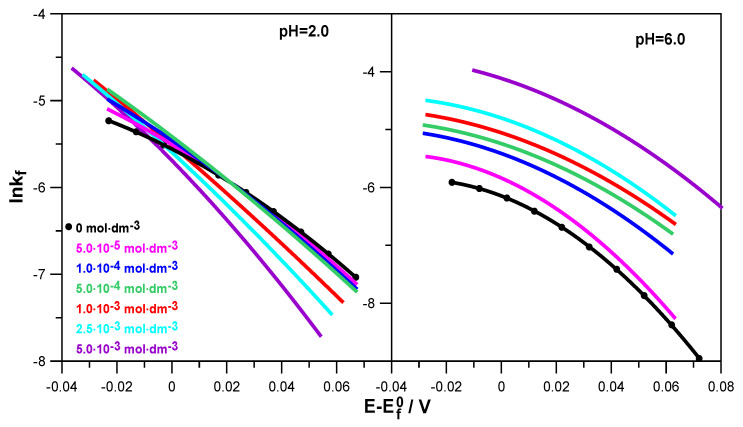
Tafel dependencies determined for the electroreduction of Zn^2+^ ions in 1.0 mol·dm^−3^ NaClO_4_ at various concentrations of L-DOPA in solutions of pH = 2.0 and pH = 6.0.

The obtained Tafel dependencies for solutions with different pHs are presented in Figure 6.

Based on them, it can be seen that lnk_f_ = f(E − E^0^_f_) was not linear and was characterized by a change in slope with the changes in electrode potential and levodopa concentration. This applied to both solutions with pH = 2.0 and pH = 6.0, although in a less acidic solution, this behavior was much more pronounced. This indicates the stepwise nature of the electroreduction of Zn^2+^ ions in 1.0 mol·dm^−3^ NaClO_4_ and in solutions containing L-DOPA. This stepwise nature probably resulted from subsequent reactions related to the exchange of charge between the electrode and the depolarizer ions but also from the preceding chemical reactions related to the dehydration of Zn^2+^ and Zn^+^ aquaions, as well as the reaction of the formation of an active complex: depolarizer ions–organic substance adsorbed on the electrode surface. The stages related to water loss and the formation of active complexes in the multistage electroreduction of Zn^2+^ ions occurred much faster than the charge transfer stages [26,27]. Based on a simple theoretical model, it can be assumed that the electroreduction of Zn^2+^ on the mercury electrode, in particular in solutions with pH = 6.0, was carried out in two stages of transferring successive electrons [28]. Of course, these stages were preceded by the above-mentioned fast chemical reactions.

During the analysis of Figure 6, it can also be seen that in a solution with pH = 2.0, an increase in the concentration of L-DOPA caused the k_f_ values to decrease in a significant part of the potential range, compared to those obtained in the absence of the drug (black curve). The higher the concentration of L-DOPA was, the greater this decrease was. The inhibitory effect of levodopa on the electroreduction of zinc ions resulted from the blocking of the electrode surface by the L-DOPA adsorbed on it. It can be assumed that the adsorption of levodopa was facilitated by its cationic form, in which it occurred next to the zwitterion form in a solution with pH = 2.0. As a result, blocking the electrode led to an increase in the overpotential and made it difficult for positively charged zinc aquaions to approach the electrode surface [21]. Different and definitely more pronounced changes in the kinetics of the electroreduction of Zn^2+^ ions were caused by the adsorption of zwitterions in the form of which L-DOPA occurred in a solution at pH = 6.0. In the entire potential range analyzed, levodopa catalyzed the electrode reaction. The acceleration effect was greater the higher the drug concentration was. It can be assumed that the diffusion of depolarizer ions to the electrode was facilitated by the attractive interaction of the acetate group of levodopa adsorbed on the electrode surface. It is likely that in specific conditions prevailing on the electrode, the presence of Zn^2+^ ions, which were in the immediate vicinity of the adsorption layer, promoted the transfer of H^+^ ions from the NH_3_^+^ group to the CH_3_COO^−^ ion, thus unblocking the free electron pair of the nitrogen atom in the NH_2_ group. Thanks to this, it was possible to create an active complex on the mercury surface: zinc ions–levodopa, mediating and facilitating the exchange of charge during the electrode process.

**Figure 7:**
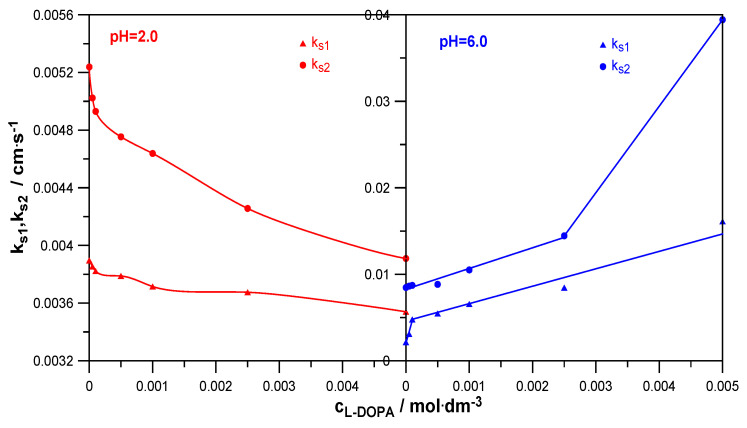
The dependencies k_s1_ = f(c_L-DOPA_) and lnk_s2_ = f(c_L-DOPA_) at different pHs.

The measures of the catalytic/inhibitory capacity of the analyzed organic substances for the subsequent stages of the electrode process were the standard stepwise rate constants k_s1_ and k_s2_ [25]. The change in their values with the increase in the L-DOPA concentration is shown in Figure 7.

As can be seen, in a solution with pH = 2.0, the presence of L-DOPA inhibited both the first and second stages of Zn^2+^ electroreduction. In a solution with pH = 6.0, both stages of the electrode reaction were accelerated. What made the influence of L-DOPA on the kinetics in both solutions similar was the fact that in both the lower and higher pH solutions, the first stage was slower and determined the speed of the entire electrode process. As already mentioned, it can be assumed that the first stage was associated with the exchange of the first electron between hydrated Zn^2+^ ions and the mercury electrode. The second stage involved the exchange of another electron between the less hydrated Zn^+^ ions and mercury. As is known, water molecules, both those from the hydration sphere of the depolarizer ions and those adsorbed on the electrode surface, constitute a significant barrier to zinc depolarization. The higher hydration of the Zn^2+^ aquaion compared to the Zn^+^ aquaion would explain the slower rate of the Zn^2+^ + e → Zn^+^ reaction than the rate of the Zn^+^ + e → Zn(Hg) reaction [23].

Investigating the inhibitory effect of levodopa on the rate of the first and second stages of the electroreduction of zinc ions on mercury in a solution with pH = 2.0 (Figure 7), it can be seen that it was definitely smaller than its catalytic effect in a solution with pH = 6.0. The introduction of levodopa to a solution with a lower pH caused a slight decrease in the values of k_s1_ (1.1-fold) and k_s2_ (1.3-fold). In turn, the presence of the drug in a solution of NaClO_4_ with pH = 6.0 caused a significant increase in the values of the standard rate constants of both stages. It should be noted here that both in the case of changes in the values of k_s1_ = f(c_L-DOPA_) and k_s2_ = f(c_L-DOPA_), two areas of linearity of these dependencies can be distinguished. Their occurrence proves the formation of various unstable active complexes on the mercury surface, which, according to a bridging model, facilitated the transfer of electrons across the inner layer [29]. They were formed when the hydration sphere of Zn^2+^ ions was partially degraded and therefore had a different composition. Further considering the change in the values of standard rate constants with the increasing concentration of L-DOPA in a solution of pH = 6.0, it can be seen that its influence on the transfer of the first electron was greater than on the transfer of the second electron. The values of k_s1_ increased 7.5-fold at the highest drug concentration, and the values of k_s2_ increased almost 5-fold. This meant that active complexes of depolarizer ions with the accelerating substance were already formed before the exchange of the first electron and were also formed before the exchange of the next electron [30].

### 2.3. Molecular Dynamics Simulations

There is no doubt that experimental research is a basic tool in the hands of scientists. However, as in every case, as a complementary, it is worth reaching for theoretical methods. They often allow for a more detailed explanation of observed phenomena and sometimes point to new directions of studies.

To expand the knowledge of investigated systems, theoretical investigations of levodopa aqueous solutions were performed by means of molecular dynamics simulations using Gromacs 2024.04 suite [31]. Calculations were conducted by applying General Amber Force Field (gaff), and the partial atomic charges were calculated using the RESP procedure [32].

In the experimental part of this paper, we investigated levodopa solutions at pH = 2.0 and pH = 6.0. Frequently, the pH solution affects the degree of dissociation of dissolved electrolytes. This was also the case here. Frazao et al. [6] showed the pH-dependent population of L-DOPA protonated states. The published research results showed that at pH = 6.0, levodopa existed only in the zwitterion dissociated form (see Figure 1b), whereas at pH = 2.0, it was present in the solution in two states, with a population of 1:1 (Figure 1a,b) [6].

Initially, simulations were performed in a box cube with an edge length of 27 nm. We prepared a few boxes corresponding to different pH and levodopa concentrations: 0 M, 5.0 × 10^−3^ mol·dm^−3^, and 5.0 × 10^−4^ mol·dm^−3^ for pH = 2.0 and pH = 6.0. The selection of L-DOPA concentrations was determined by its ion number in the simulation box. In the case of its lowest concentration of 5.0 × 10^−4^ mol·dm^−3^, the applied box with an average volume of 19.683 nm^3^ included only four L-DOPA ions. If one wants to simulate solutions with its lower concentrations, the simulation box should be significantly enlarged, which will cause a high increase in simulation times.

Taking into account the research published by Frazao et al. [6], systems with pH = 6.0 included only L-DOPA state (b), the “zwitterion anion”, whereas systems with pH = 2.0 were prepared by using equal numbers of both L-DOPA states, as shown in Figure 1.

Simulation systems reflected the composition of solutions used in the experiment. Therefore, besides L-DOPA ions, boxes included adequate amounts of tip3p water, ClO_4_^−^, NO_3_^−^, Zn^2+^, and Na^+^ ions. Calculations were performed in the isothermal–isobaric ensemble at T = 298.15 K and *p* = 1 bar applying the Nose–Hoover thermostat and the Parrinello–Rahman barostat. For long-range electrostatic interactions, we used the PME algorithm. Lennard-Jones and columbic cut-off distances were set to 1.2 nm. Equations of motion were solved using the Verlet leap-frog algorithm with a time step of 1 fs. Each simulation was initially equilibrated for 20 ps and then run for 2.5 ns.

**Figure 8:**
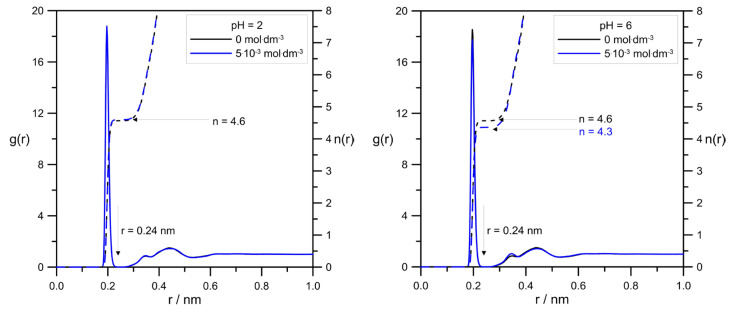
The effect of L-DOPA concentration on RDFs calculated for pair Zn^2+^/tip3p water at different pHs. The values of the number of water molecules in zinc hydration sphere were determined by assuming its radius to be r = 0.24 nm.

One of the most common and useful tools to analyze simulation data is the radial distribution function g(r) (RDF). Figure 8 shows the comparison of RDFs calculated for the pair Zn^2+^/tip3p water for systems with varied levodopa concentrations at pH = 2.0 and pH = 6.0.

**Figure 9:**
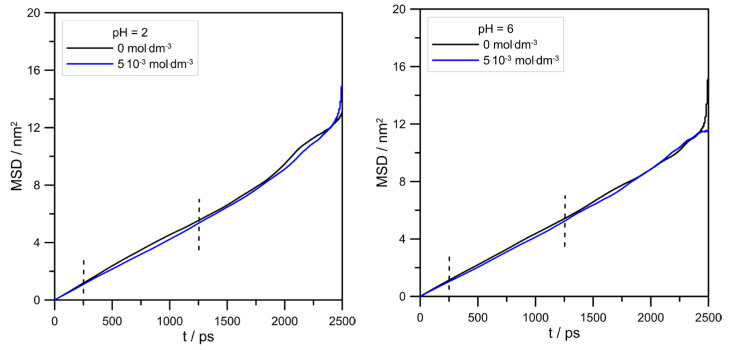
The effect of the L-DOPA concentration on the Zn^2+^ mean square displacement at different pHs. Vertical, dashed lines show the range of MSD used for linear regression (from 250 to 1250 ps).

Figure 9 shows a very interesting effect of L-DOPA on the solvation sphere of Zn^2+^ ions. First of all, at the acidic pH = 2.0, the highest L-DOPA concentration had no effect on the number of water molecules in the zinc hydration sphere. In the case of pH = 6, this effect was visible: the increase in the levodopa concentration caused a decrease in the number of tip3p water in the zinc hydration sphere. What is more, pH and L-DOPA did not affect the radius of the hydration sphere. With the increasing distance between Zn^2+^ and tip3p water, RDFs were at both pHs identical, and they were alike for 0 mol·dm^−3^ and 5.0 × 10^−3^ mol·dm^−3^ levodopa concentrations as well.

The influence of L-DOPA on the mobility of zinc ions could be reflected by its mean square displacement (MSD). MSD is the measure of particle travel following a random walk plotted as the function of time. In addition to that, MSD is related to self-diffusivity D of a particle by the following formula:

(1) $$D=\frac{1}{2d}\underset{t\to \infty }{\lim}\frac{d}{dt}MSD\left(r\right)$$

where r is the displacement, and d is the dimension of a system. Strictly speaking, the diffusion coefficient can be determined from the tangent of the MSD slope divided, in our case, by six. Figure 9 and Table 1 show MSD curves determined for simulated systems and calculated zinc diffusion coefficients.

The values of Zn^2+^ diffusion coefficients were in agreement with the conclusion drawn using RDFs, as shown in Figure 8. In general, the hydration level of the zinc cation should affect its mobility in an aqueous solution. Because at pH = 2.0, the cationic L-DOPA form did not affect Zn^2+^, the hydration number (tip3p water molecules in its hydration sphere), the value of the Zn^2+^ diffusion coefficient was approximately unchanged (see Table 1). At pH = 6.0, the number of water molecules in the Zn^2+^ hydration sphere decreased from 4.6 to 4.3, and the Zn^2+^ diffusion coefficient value slightly increased. However, diffusion coefficients determined from MSD were burdened with error, as shown in Table 1, and the above conclusion can be drawn only when Zn^2+^/tip3p RDFs calculations were taken into account.

**Figure 10:**
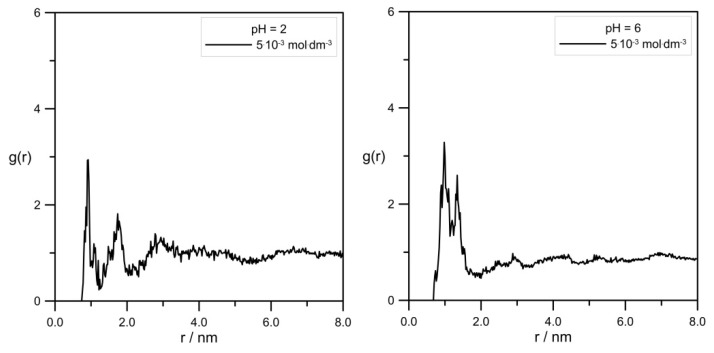
RDFs calculated for the pair L-DOPA/Zn^2+^ at different pH and levodopa concentrations of 5 × 10^−3^ mol·dm^−3^.

MD calculations enabled us to draw a more precise view of the average structure of simulated systems. Figure 10 shows pair correlation functions determined for the L-DOPA/Zn^2+^ pair.

**Figure 11:**
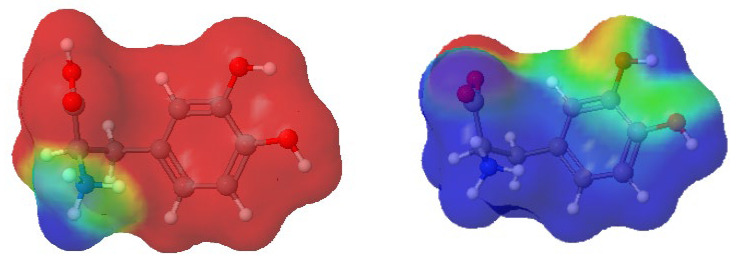
Charge distribution in L-DOPA ions: protonated (**left**) and zwitterion (**right**). Red color denotes a negative charge, whereas blue means a positive one. The partial charges on atoms were calculated using the RED Server Development service, which produces the so-called resp charges [33].

First of all, it should be emphasized again that simulation systems contained a small amount of Zn^2+^ and L-DOPA molecules, and RDF curve shapes shown in Figure 10 were far from perfect. Nevertheless, it can be seen that the distance between L-DOPA and the Zn^2+^ ions was affected to a small extent by pH and levodopa concentrations. The most valuable data was for the 5 × 10^−3^ mol·dm^−3^ concentration because in these simulation systems, we obtained better statistics (the larger number of L-DOPA molecules in comparison to 5 × 10^−4^ mol·dm^−3^). Figure 10 shows, at pH = 2.0, two peaks at approx. 0.9 nm and 1.8 nm, which corresponded to two different ionic forms of L-DOPA. At pH = 6, we can observe one peak at about 1.1 nm. It can be suspected that the protonated L-DOPA (Figure 1a) was closer to Zn^2+^ than its zwitterion form (Figure 1b). The simultaneous presence of protonated -NH_3_^+^ and dissociated -COO^−^ groups caused a serious change in charge density in the L-DOPA molecule. As a result, the protonated L-DOPA form attracted the zinc cation more strongly than its zwitterion form. This thesis confirmed the charge distribution of both levodopa states, as shown in Figure 11. Moreover, a slight decrease in the hydration number of the Zn^2+^ at pH = 6 also made it easier to get levodopa closer to the zinc cation.

**Figure 12:**
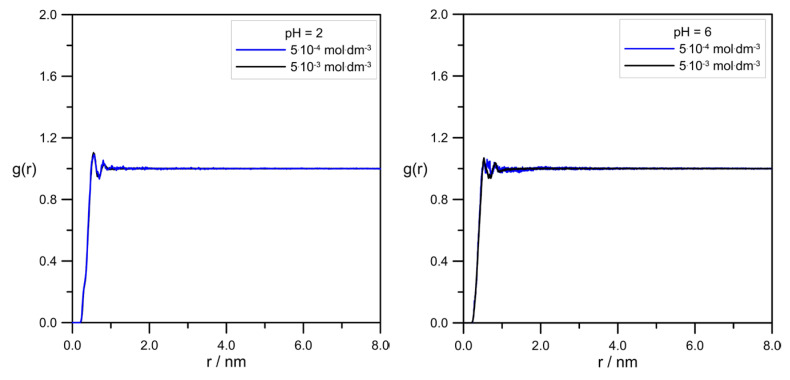
RDFs calculated for the pair L-DOPA/tip3p at different pH and levodopa concentrations.

Figure 12 demonstrates the comparison of pair correlation functions for the L-DOPA/tip3p pair.

The radial distribution functions in Figure 12 were almost identical for all simulation systems. It indicated the lack of influence of pH and levodopa concentrations on the average mutual arrangement of levodopa and water. Minimal differences in L-DOPA/tip3p RDF shapes at pH = 6.0 were probably caused by the presence of the zwitterion form of the levodopa ion.

## 3. Discussion

The analysis of the differential capacitance curves obtained for the analyzed solutions at pH = 2.0 and pH = 6.0 indicates the labile adsorption of levodopa on the mercury electrode in the potential range of the electroreduction of Zn^2+^ ions. It can therefore be expected that L-DOPA adsorbed in the zwitterion and protonated forms at both pH = 2.0 and pH = 6.0 influenced the kinetics of the analyzed electrode reaction. CV and SWV voltammetry methods and the EIS impedance method allowed for a qualitative description of this influence. It was shown that the increase in L-DOPA concentration in the Zn^2+^ ion solution at pH = 2.0 caused a gradual small decrease in the SWV peak current, an increase in the value of the difference between the anodic and cathodic peak potentials (∆E) in the CV voltammograms, and an increase in the activation resistance (R_a_) value determined from the EIS spectra. Such trends of changes indicate a slight inhibiting effect of the drug on the electrodeposition of zinc on mercury from solutions with pH = 2.0. Completely different trends of changes in the parameters analyzed above were observed in solutions with pH = 6.0. The increase in the L-DOPA concentration resulted in a significant increase in the SWV peak heights and a decrease in ∆E and R_a_ values. These changes proved a significant accelerating effect of L-DOPA on the kinetics of the analyzed electrode process. The catalytic effect of L-DOPA in solutions with pH = 6.0 increased with the growth of the drug concentration.

Precise analysis of the drug’s effect on the rate of Zn^2+^ ion electroreduction on the mercury electrode was possible based on the calculated rate constants’ values as a function of the electrode potential and the standard rate constants’ values of the subsequent electroreduction stages. As it resulted from the nonlinear Tafel dependencies obtained for solutions at pH = 6.0, the electrode reaction, both in the absence and presence of L-DOPA, had a stepwise nature associated with the exchange of successive electrons but also with chemical reactions. Chemical reactions involved the dehydration of Zn^2+^ and Zn^+^ aquaions and the formation of unstable active complexes on the electrode surface between the adsorbed L-DOPA zwitterions and the depolarizer ions. These complexes facilitated the exchange of charge during the electrode process. The obstacles to its implementation were water molecules adsorbed on the mercury surface and the hydration sphere of the depolarizer ions. Therefore, the L-DOPA-Zn^2+^ and L-DOPA-Zn^+^ complexes formed in the specific conditions prevailing on the electrode (cap-pair effect) constituted a pathway that allowed the minimization of the above-described “water” obstacles. Moreover, a beneficial factor in accelerating the electrodeposition of zinc on the mercury electrode was the presence of the L-DOPA zwitterion state in solutions with pH = 6.0. As shown by molecular dynamics simulations, an increase in the concentration of zwitterion L-DOPA reduced the average number of water molecules in the hydration sphere of zinc in solutions with pH = 6 and thus slightly facilitated the diffusion of zinc to the electrode. Simultaneously determined from MD simulations, the Zn^2+^ diffusion coefficient value was a little bit larger than in the case of the solution without L-DOPA. This effect did not occur in solutions with pH = 2.0. The simultaneous presence of the protonated form and zwitterion L-DOPA in solutions with pH = 2.0 did not affect the hydration sphere of depolarizer ions. The inhibition effect of the electroreduction of Zn^2+^ ions by L-DOPA in solutions with pH = 2.0 described above resulted from the blocking of the electrode surface by both forms of the drug adsorbed on it. Probably also in the solutions with low pH, unstable active complexes of depolarization ion-organic compounds had a chance to be formed. Nevertheless, the effect of blocking the electrode surface was dominant over the cap-pair effect, and finally, in the solutions at pH = 2.0, an inhibiting effect of L-DOPA was observed.

## 4. Materials and Methods

In the experimental part, the solution of 5.0 × 10^−3^ mol·dm^−3^ Zn^2+^ in 1.0 mol·dm^−3^ NaClO_4_ used as the supporting electrolyte served as a reference solution. Solutions were analyzed at pH = 2.0 and pH = 6.0. pH solutions were adjusted using an Orion Star A211 pH meter (Thermo Fisher Scientific, Waltham, MA, USA). L-DOPA was added in concentrations ranging from 5 × 10^−5^ mol·dm^−3^ to 5 × 10^−3^ mol·dm^−3^. The solutions were freshly prepared just before the measurements and deaerated by purging nitrogen over them during the measurements.

The following analytically pure reagents were used in the studies: Zn(NO_3_)_2_·6H_2_O (Fluka), NaClO_4_, and levodopa (Sigma-Aldrich, Burlington, MA, USA). All solutions were prepared with deionized water generated using a Milli-Q water purification system (Millipore, London, UK).

The measurements were carried out using an electrochemical analyzer μAutolab/FRA 2 (Eco Chemie, Utrecht, The Netherlands) with GPES v. 4.9.007 software. The analyzer was used in conjunction with the controlled growth mercury drop electrode (CGMDE, M165, MTM-ANKO, Warsaw, Poland), which functioned as either a dropping electrode (DME) or a hanging electrode (HMDE). The measurements were performed in a three-electrode system comprising a working electrode (mercury electrode with a droplet surface area of 0.013677 cm^2^), a reference electrode (Ag/AgCl with a saturated NaCl solution), and an auxiliary electrode (platinum spiral).

In studies of electrode process kinetics, voltammetry (SWV and CV), DC polarography, and electrochemical impedance spectroscopy (EIS) were used. The optimal working conditions for each method were as follows: pulse amplitude of 10 mV, frequency of 10 Hz, and step potential of 10 mV (for SWV voltammetry); step potential of 2 mV and scan rate of 100 mV·s^−1^ (for CV voltammetry); and step potential of 10 mV (for DC polarography). Impedance data was collected at 36 frequencies, ranging from 15 to 100,000 Hz, in the Faradaic potential region at 10 mV intervals.

The AC impedance technique (frequency 800 Hz, amplitude 5 mV) was used to study adsorption at the Hg/L-DOPA solution interface.

The measurements were carried out at a temperature of 298 ± 0.1 K.

## Figures and Tables

**Table 1:** The values of Zn^2+^ diffusion coefficients determined from MD simulations of investigated systems applying the Einstein Equation (1).

Diffusion Coefficient/10^−5^ × cm^2^·s^−1^		c_L-DOPA_/mol·dm^−3^
pH = 2.0	pH = 6.0	
0.7025 (±0.10)		
0.7162 (±0.01)	0.6981 (±0.01)	5 × 10^−3^

## Data Availability

Data are available upon request.
